# A highly stable minimally processed plant-derived recombinant acetylcholinesterase for nerve agent detection in adverse conditions

**DOI:** 10.1038/srep13247

**Published:** 2015-08-13

**Authors:** Yvonne J. Rosenberg, Jeremy Walker, Xiaoming Jiang, Scott Donahue, Jason Robosky, Markus Sack, Jonathan Lees, Lori Urban

**Affiliations:** 1 PlantVax Inc, Rockville, MD, 20850; 2 FLIR Systems Inc, Pittsburgh, PA, 15238; 3 Institute for molecular Biotechnology, RWTH Aachen University, Aachen, Germany

## Abstract

Although recent innovations in transient plant systems have enabled gram quantities of proteins in 1–2 weeks, very few have been translated into applications due to technical challenges and high downstream processing costs. Here we report high-level production, using a *Nicotiana benthamiana/p19 system*, of an engineered recombinant human acetylcholinesterase (rAChE) that is highly stable in a minimally processed leaf extract. Lyophylized clarified extracts withstand prolonged storage at 70 °C and, upon reconstitution, can be used in several devices to detect organophosphate (OP) nerve agents and pesticides on surfaces ranging from 0 °C to 50 °C. The recent use of sarin in Syria highlights the urgent need for nerve agent detection and countermeasures necessary for preparedness and emergency responses. Bypassing cumbersome and expensive downstream processes has enabled us to fully exploit the speed, low cost and scalability of transient production systems resulting in the first successful implementation of plant-produced rAChE into a commercial biotechnology product.

Nerve agents and pesticides cause toxicity by inhibiting the activity of acetylcholinesterase (AChE) in neuromuscular junctions and in blood^1^,^2^. Due to their high reactivity with organophosphate (OP) compounds, cholinesterases such as AChE and butyrylcholinesterase (BChE) are being evaluated both as therapeutic human pretreatments^3^,^4^,^5^,^6^,^7^ and for their effectiveness in enzyme-based detection and decontamination/neutralization technologies for remediation purposes, for example following accidental or deliberate release of chemical warfare agents (CWA)^8^. Recombinant (r)AChE can find application in field-portable diagnostic assays, and in sensors to determine the location of chemical agents on surfaces with the intent to decontaminate the surface. However, physiologically active AChE would denature and not persist for sufficient time to complete decontamination, thus a highly thermally stable version of ChE is a high priority for use as a catalytic reporter for detection of nerve agents on hot surfaces and at elevated temperatures such as desert conditions.

Recombinant AChE and BChE capable of sequestering OPs *in vivo* have been successfully produced in transgenic goat milk, mammalian cells and plants^9^,^10^,^11^,^12^. Another OP bioscavenger, organophosphate hydrolase (OPH), has also been produced stably in maize^13^ and tobacco plants^14^ and transiently in tomato fruit and *N.benthamiana* (unpublished) for environmental OP bioremediation, as well as therapeutic purposes. However to date, none of these approaches has yet translated into a product because of low expression levels, insufficient thermal stability for certain intended applications and/or challenges and costs in downstream processing.

Here we demonstrate that the demanding criteria associated with rapid and cost effective product development have been successfully addressed using an *Agrobacterium*-mediated transient co-expression system in *N. benthamiana (N.b.)* plants to produce highly stable rHuAChE in a minimally processed extract form at levels that ensure product effectiveness and economic viability. The initial challenges of low expression and stability of the rHuAChE were overcome in part by co-transfecting three different genes in 6–8 week old *N.b*. plants: (i) a codon optimized glyco-engineered (D^**61**^N and S^**541**^N) sequence (originating from E32) for the hydrophilic AChE-E4-E6 isoform (also described as AChE-E6^15^), in which exon 4 is joined to exon 6, permitting the formation of tetramers (ii) the p19 suppressor of gene silencing from tomato bushy stunt virus for increasing expression levels^16^ and (iii) the proline-rich attachment domain (PRAD) of the Colq gene encoding a 17-residue peptide at the N terminus of the AChE collagen tail, which further optimized assembly into tetramers and significantly increased overall expression levels^17^. The addition of PRAD raised levels of rHuAChE from 180 mg/kg to 700–900 mg/kg when harvested at 9–12 days after infiltration: presumably a result of stabilizing newly synthesized AChE subunits in the endoplasmic reticulum (ER)^18^ and up-regulating the number of tetrameric molecules exiting the ER and entering the golgi and other compartments.

Since it was initially assumed that OP detection devices would require purified enzyme, *N.b/p19*-derived rAChE-containing extracts were purified using procainamide Seph-arose chromatography and tested for kinetic properties and stability. Figure 1a shows that both the purified and extract forms exhibited the same inhibition rate constants (ki) and were equivalent to or better than two non-plant-expressed, in-house controls against nine different OP pesticides and diisopropylfluorophosphate (DFP). Purified tetrameric rAChE at 700 U/ml (200 ug/ml) also exhibited high stability with and without supplemental collagen when incubated at 4 °C, RT (23 °C) and 37 °C, with <20% loss of activity after 21 days at 37 °C (Fig. 1b). Surprisingly, further testing of the crude extract and purified forms held at 40 °C for 8 hr, at a concentration similar to that used in the detection devices (16 U/ml = 5 ug/ml), indicated that the rAChE in extracts appeared to be protected from thermal denaturation; exhibiting stability in the absence of the stabilizers (collagen, N-acetyl l-cysteine (NALC) and ethylenediamine tetraacetic acid (EDTA)) typically used to maintain activity of purified rAChE (Fig. 1b). The excellent properties of the rAChE plant extracts as well as the high rAChE expression levels changed the focus of the study to optimizing the processing of unpurified extract such that it can be effectively lyophilized and stored continuously at 70 °C for 6 weeks without loss of activity, in order to meet the military’s stringent operational requirements for OP detection devices. Figure 2 illustrates the key steps in the overall process and product development, and shows various modes in which the final extract-based product can be used in sensor applications.

One of the most important challenges commonly encountered when working with plant extracts is the presence of polyphenols (PP) and polyphenol oxidase (PPO) which lead to “browning” that can cause loss of protein activity and complicate subsequent purification^19^,^20^. Although PP can be advantageous in “turning on” *Agrobacterium* virulence genes that help to infect and transfer plasmids to plant cells^21^, they can subsequently result in detrimental pigment formation during storage at high temperatures. Several additives, including those used at an industrial scale by the food and beverage industries to enhance product appearance and quality, were tested for their ability to suppress the browning reaction both during processing of rHuAChE extracts and stabilization of the dried product. Of many additives tested, three were found to have the most beneficial effects on the quality and activity of rHuAChE extracts during processing and lyophilization; addition of polyvinylpyrrolidone (PVP) and sodium metabisulphite to absorb and prevent formation of polyphenols, and the addition of chitosan to remove oils and fats. Figure 2a shows the effective clarification of the initial homogenized leaf pulp after addition of 3% w/v PVP and 0.2% v/v chitosan (Fig. 2a-1) followed by subsequent centrifugation (Fig. 2aa-2). The holes in the rack holding the sample can be easily observed through the clarified extract. Lyophilization of this extract resulted in a cream colored powder (Fig. 2a-3) that was fully and easily reconstituted without any loss of enzyme activity when assessed either immediately (Fig. 2b-1) or after 6 weeks at 70 °C (Fig. 2b-2). By contrast, extract lacking PVP turned brown and lost AChE activity after 6 weeks at 70 °C (Fig. 2b-3).

The high thermal stability of plant-rHuAChE is attributed to the level of oligomerization of the AChE molecules (which renders them more heat resistant), and is consistent with the finding that plant-derived monomeric rAChE does not exhibit the high thermal stability observed with the tetrameric form^22^. The oligomerization in this case is a consequence of co-expression of both the AChE and PRAD genes and potentially an increase in the number of glycosylation sites on the mutated E4 molecule. Although the stability of the aqueous extract at 37–40 °C was very good in the absence of collagen (Fig. 1), collagen was added to the dry powders primarily because of its high glass transition temperature (T_g_); effectively bulking the enzyme powder for reliable handling and increasing the crystallinity of the enzyme powder, preventing phase separation which may occur when powder is stored at elevated temperatures. This strategy has been previously used to stabilize pharmaceuticals and has been exploited to improve rHuAChE stability for these technologies as well.

Having established the stability in both liquid and powdered forms, the enzymatic function of the reconstituted rHuAChE powders was tested in military field detection technologies (sprays and point sensors), initially on tiles, and subsequently on other surfaces. These sensors utilize AChE, which produces acetic acid when it hydrolyzes acetylcholine, along with base-producing enzymes that generate ammonia to drive a chemical reaction that dynamically buffers the system pH. Thus, when AChE is inhibited by an OP compound, acetic acid production shuts off, and the base-producing enzymes continue to make ammonia driving the system pH dramatically from 5 to 8. A colorimetric pH-responsive dye (pKa 6.5) is in turn titrated from yellow to red, providing a localized visual assessment of the presence of the OP. It should be noted that, considering the high specific activity of AChE towards acetylcholine, these sensors require a miniscule concentration of enzyme, which once dissolved for use equates to <1 mg/mL protein; a concentration that does not contribute sufficient buffer capacity to impede the response.

For testing the limits of OP detection on tiles, serial dilutions of the OP paraoxon (Px) were prepared in isopropyl alcohol such that 10 ul delivered 10, 5, 1, 0.5, and 0.1 ug of Px. Five spots at each concentration were then applied to a tile at RT, or one that has been chilled or heated to temperatures ranging from 0 °C to 50 °C, and then sprayed with a mixture containing a pH-sensitive yellow dye, enzyme and substrate. A change in color to pink/red indicates the presence of Px. Figure 2c-1 shows a tile at RT with the limit of detection of 0.1 ug Px while Fig. 2c-2, 2c-3 indicate dete**c**tion of single 10 ug Px spot after spraying on tiles at 50 °C and 0 °C respectively.

Several sensing devices have recently been developed that can effectively utilize the plant-derived rHuAChE to detect trace quantities of both G- & V-series OP nerve agents on surfaces and provide the US military the capability to visually identify areas where OP is present on vehicles or on personal protective equipment. These can be used in wide areas, to enable more rapid, focused decontamination. Enzymatic technologies such as these, which can detect agent directly on the surface, are potentially more sensitive than current state-of-the-art electronic devices, which only detect agent vapors. They would enable detection of both volatile (sarin, soman) and low-volatility OPs, such as VX, directly on surfaces. In the current studies, Px, a structural analogue of nerve agents, has been used to assess the activity of the rHuAChE plant extracts. Px is a phosphorate, and most of the nerve agents are methyl phosphonates (sarin, soman, cyclosarin, VX and VR) or a cyanophosphoramidate (tabun). All of these compounds, whether they be a pesticide “oxon” metabolite or a nerve agent, show similar OP inactivation kinetics, a propensity to age and susceptibility to oxime reactivation^23^,^24^. Given the similarity in hydrolytic mechanisms, Px has been shown to be a suitable marker for detection of OPs by enzymatic hydrolytic susceptibility.

Using OP-sensing chemistry Fig. 2c, several types of devices have been developed to provide the user with different methods for identifying nerve agent contamination on surfaces. In the first case, the chemistry was implemented within a two-component liquid spray, the Agentase Disclosure Spray (Fig. 2d-1, which can be applied directly to surfaces such as terrain (Fig. 2d-2), roads (Fig. 2d-3), and military vehicles. The enzymatic components, including the plant-based AChE, are kept in one liquid reservoir, while enzyme substrates and other additives (tinting compounds, surfactants, rheological thickeners and enzyme substrates) are kept in a second reservoir. The spray is applied from a special applicator that mixes the two components in equivalent ratios at the nozzle, as the solutions are dispensed.

In the Chemical Agent Detection (CAD) Pen, the AChE was embedded in a polyurethane foam in a pen-like point sampling device. The barrel of the device contains dried chemistries and a glass ampule full of aqueous buffer. The user cracks the ampule to activate the device, then inverts the pen and turns the barrel to introduce the wet chemistry to the enzymatic foam. Once activated, the cap is removed and the sponge can be used to sample surfaces. Once a surface is sampled, the cap is replaced, and the colorimetric scheme reports on whether there are any OPs present within two to five minutes (Fig. 2d-4). Both the spray and the pen, due to their use by the military, have stringent enzyme quality requirements and must possess extended shelf-life times for the products in field temperatures exceeding 37 °C. The minimally-processed recombinant plant-expressed AChE proved to be a viable, low-cost replacement for prior rAChE sources, and now serves as a model for future economical methods for producing proteins in biotechnology products.

The development of new transient plant expression systems for recombinant protein production, offers the advantages of rapid, cost effective, large scale production and adaptability, without the fear of human pathogens; resulting in the re-evaluation of the potential of molecular farming^25^,^26^. The bottlenecks that have prevented more pharmaceutical and non-pharmaceutical plant-based biologics from becoming commercial realities have been the costs associated with down-stream purification steps and regulatory approval. In addition to rapid, high level expression, the present study has demonstrated that, by combining molecular engineering to enhance oligomerization and efficient processing of extracts to eliminate oils, fats and PP, the transient *N.b/p19* system can produce a highly thermally stable minimally processed rHuAChE extract that does not lose activity over a 70 °C range and can be formulated for use in newly developed devices for the detection of organophosphate nerve agents and pesticides. Indeed, such extracts may have an advantage over purified enzyme in that they appear to constitutively contain components that prevent AChE denaturation at high temperatures, without interfering with the sensitive pH-dependent readout of the devices. Elimination of the down stream processing steps will significantly reduce time to market and cost of rHuAChE produced at larger scales (estimated to be in excess of 200 MU per year). We have also recently produced rHuAChE in field-grown *N. tabacum plants* at 100 mg/kg, which offers an efficient large-scale alternate means of scaling up production.

## Methods

### Plant Expression Vectors

The DNA sequence encoding the splice variant AChE E4–E6 isoform (Accession #P22303) with the D^**61**^N and S^**541**^N mutations was optimized for *Nicotiana benthamiana* expression, synthesized by GenScript (Piscataway, NJ) and inserted into the T-DNA vector pTRAk using *Eco*RI and *Bam*HI sites. The plasmid was verified by DNA sequencing. The AChE plasmid was co-transfected with two additional pTRAk constructs. The first one is pTRAk-PRAD, which contains the first 67 amino acid of rat acetylcholinesterase-associated collagen (COLQ) (AF007583). The second one is pTRAk-TBSV containing the tomato bushy stunt virus p19 inhibitor of silencing (AJ288917).

### Agrobacterium, transient transfection and plant cultivation

Transformation, selection and cultivation of *Agrobacterium tumefaciens* strain GV3101 (pMK90RK) and cultivation *Nicotiana benthamiana* plants in the greenhouse or in growth chambers was essentially performed as described previously^16^. Briefly, recombinant Agrobacteria were cultivated in YEB medium with appropriate antibiotics, pelleted and resuspended in MS medium to OD600 nm = 0.5 and infiltrated either by injection or vacuum using 6–8 week old plants or leaves of *N.benthamiana*. For co-infiltration experiments, Agrobacteria carrying the AChE, PRAD and p19 gene expression plasmids were mixed in the ratio of 2:1:1 immediately before use. After infiltration, plants were incubated at 20 °C with a 16/8 hr day-night cycle for up to 16 days. For initial screening, six leaf discs (~11 mg) were collected from different positions in transfected leaves, ground in 200 ul 50 mM phosphate buffer, centrifuged and assayed for AChE levels from days 3 to 16. Leaves were then either directly harvested and processed or stored at −20 °C; a small 200 g batch of leaf biomass typically producing ~70 mg of rAChE.

### Preparation of Extract

One liter of extraction buffer was prepared by adding 5 mM MgCl_2_, 4 mM DTT, 5 mM sodium metabisulfite and 10% (w/v) sucrose to PBS pH 7.4. Polyvinylpyrrolidone (PVP) 40,000 MW was then added at 3% w/v and mixed until completely dissolved. Buffer was subsequently chilled at 4 °C. Frozen leaves were ground in a Vitamix blender with 5 times (w/v) extraction buffer. After grinding, the slurry was passed through Miracloth (Calbiochem #475855), and centrifuged at 20,000 *g* for 15 minutes. Chitosan was prepared (Chitosan, low molecular weight, Sigma Aldrich 448869) by adding 1% w/v chitosan into 1% acetic acid^28^. Chitosan was stirred until dissolved, at least 30 minutes, until the solution appeared gelatinous. After centrifugation, the supernatant pH was changed to 7.4 and chitosan was added at 0.2% v/v. Extract with chitosan was then stirred at 4 °C for 30 minutes, incubated for another 30 minutes without stirring, and centrifuged at 1500 rpm in a refrigerated tabletop centrifuge (Sorvall RT6000) at 4 °C for 5 minutes. Supernatant was decanted and left at 4 °C until rAChE level was determined. Extract was aliquoted and frozen at −20 °C. AChE was purified using procainamide Sepharose chromatography and eluted using either procainamide (0.2 M), decamethonium (0.02 M) or acetylcholine (0.2 M) as described previously^27^. Each resulted in similar yields and properties (data not shown).

### AChE assays

Plant-derived rAChE activity was assayed as previously described^10^. Briefly, leaf extracts or purified AChE were first diluted in 50 mM phosphate buffer containing 0.5% (w/v) BSA and 10 μl added to a plate in the presence of 1 mM ATC (Sigma Aldrich) as substrate and 1 mM DTNB. Rates of ATC hydrolysis were read at 412 nm and reported as U/ml. One unit represents 1 μmole of ATC hydrolyzed per min.

### Inhibition Assays

OP pesticide and DFP inhibitors were diluted from concentrated stocks in isopropyl alcohol with 50 mM phosphate containing 0.25 mM DTNB yielding final assay concentrations of 1 mM to 100 pM per well. Ten μL of rHuAChE diluted in buffer containing 0.4% BSA to produce a turnover rate of 100–200 mOD per minute were added to wells containing OP as well as control wells with the same isopropyl alcohol concentration. After a 10 min incubation, 1 mM ATC was added and absorbance at 405 nm was measured over 10 minutes. The ki is the ratio of the inhibited rate to the control rate for the highest inhibitor concentration at which significant enzyme inhibition occurs.

### Aqueous stability studies

Initially plant-derived AChE extracts or purified forms using different elution buffers were incubated at ~700 U/ml at 4 °C, RT and 37 °C for 3 weeks to assess stability. For these pot life studies, samples of purified rHuAChE and AChE-containing extract were diluted in 100 mM potassium phosphate pH 7 to achieve the working concentration of 16 U/ml and assessed for stability following incubation in 1 ml aliquots at 40 °C for 0–8 hours.

### OP Detection using lyophilized and reconstituted AChE

Prior to lyophilization, a mixture of collagen (0.1 mg/U) and hydroxyethylcellulose were added to the extracts to stabilize the AChE during lyophilization at a 50:1 ratio of additive to AChE. The explicit components and their concentrations in the formulations cannot be disclosed due to restrictions imposed by the International Traffic in Arms Regulations (ITAR). These technologies are defense articles used by the US Military and are on the US munitions list, and as such the details are prohibited from being fully disclosed by the US State Department. The fundamental art in the invention is generally described in US Patent 6,750,033 and in US Patent publication number US 2010/0227345 A1.

Drying of the extracts utilized a slow freeze and a standard primary/secondary drying progression with storage in a 70 °C oven in standard ADS packaging. Plastic spray bottles containing lyophilized AChE powder were back filled with nitrogen and sealed into a Mylar bag. Following incubation for different times (1–6 weeks), powders were reconstituted and tested for the limits of OP detection by serially diluting Px in isopropyl alcohol such that 10 ul delivered 10, 5, 1, 0.5, and 0.1 ug of Px. Five spots at each concentration are then applied to tiles at RT or 10 ug of Px were tested on single tiles that had been chilled or heated to temperatures from −20 °C to 50 °C. After evaporation of the alcohol, tiles were then sprayed with the yellow ADS solution containing rAChE. Spray assays were also performed after reconstituted spray was incubated for 8 hours at 40 °C or 24 hours at RT. (Note: In the two components of the ADS spray, side A contains dyes and enzyme substrate, and side B contains the enzymes such as urease, glutaminasae, creatinine amino hydrolase. These are mixed in equal ratios in the applicator nozzle during spraying). Detection of OP was determined when 4/5 spots changed to pink by five minutes. In these studies, shelf-life refers to stability of the lyophilized powder, tested immediately after reconstitution, and pot-life refers to enzyme activity over an incubation period after the powder is reconstituted.

## Additional Information

**How to cite this article**: Rosenberg, Y. J. *et al*. A highly stable minimally processed plant-derived recombinant acetylcholinesterase for nerve agent detection in adverse conditions. *Sci. Rep*. **5**, 13247; doi: 10.1038/srep13247 (2015).

## Figures and Tables

**Figure 1:**
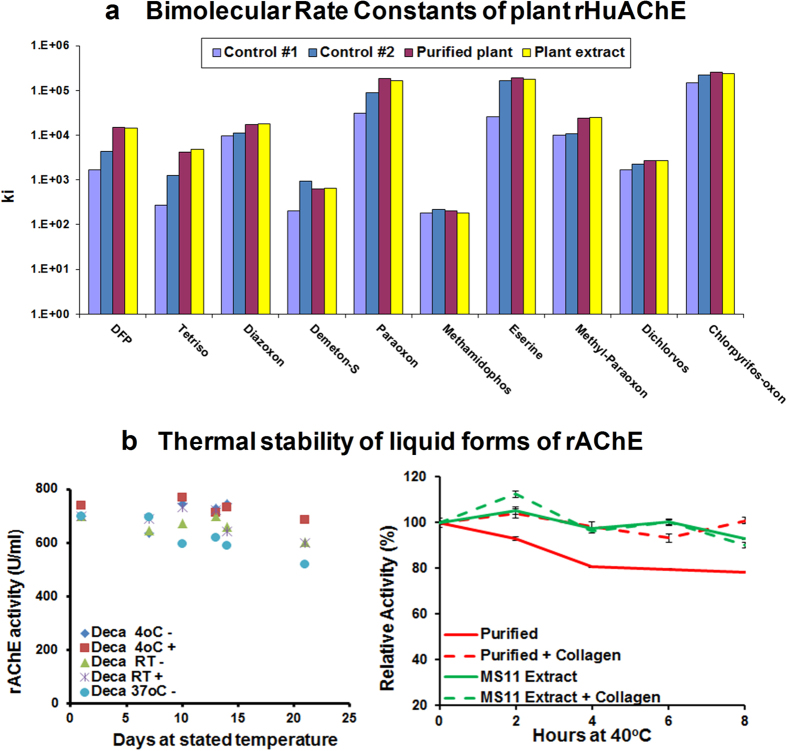
Kinetics and thermal stability of aqueous plant-derived rHuAChE (**a**) Inhibition rate constants (ki; M^−1^ min^−1^) of purified and extract forms plus two non-plant in-house rAChE controls tested against 9 OP pesticides and diisopropylfluorophosphate (DFP). (**b**) Thermal stability of purified rHuAChE (eluted with decamethonium) at 4 °C, 23 °C and 37 °C with (+) and without (−) collagen (0.1 mg/U) (left). Stability of purified and extract forms at 40 °C for 8 hr with and without collagen (right). It should be noted that the bimolecular rate constants of the plant forms of rHuAChE are similar to or better than the two non-plant controls (yeast and mushroom rHuAChE), which were previously demonstrated to show a strong correlation between bimolecular rate constants of AChE and sensor detection performance for both OP pesticides and nerve agents.

**Figure 2:**
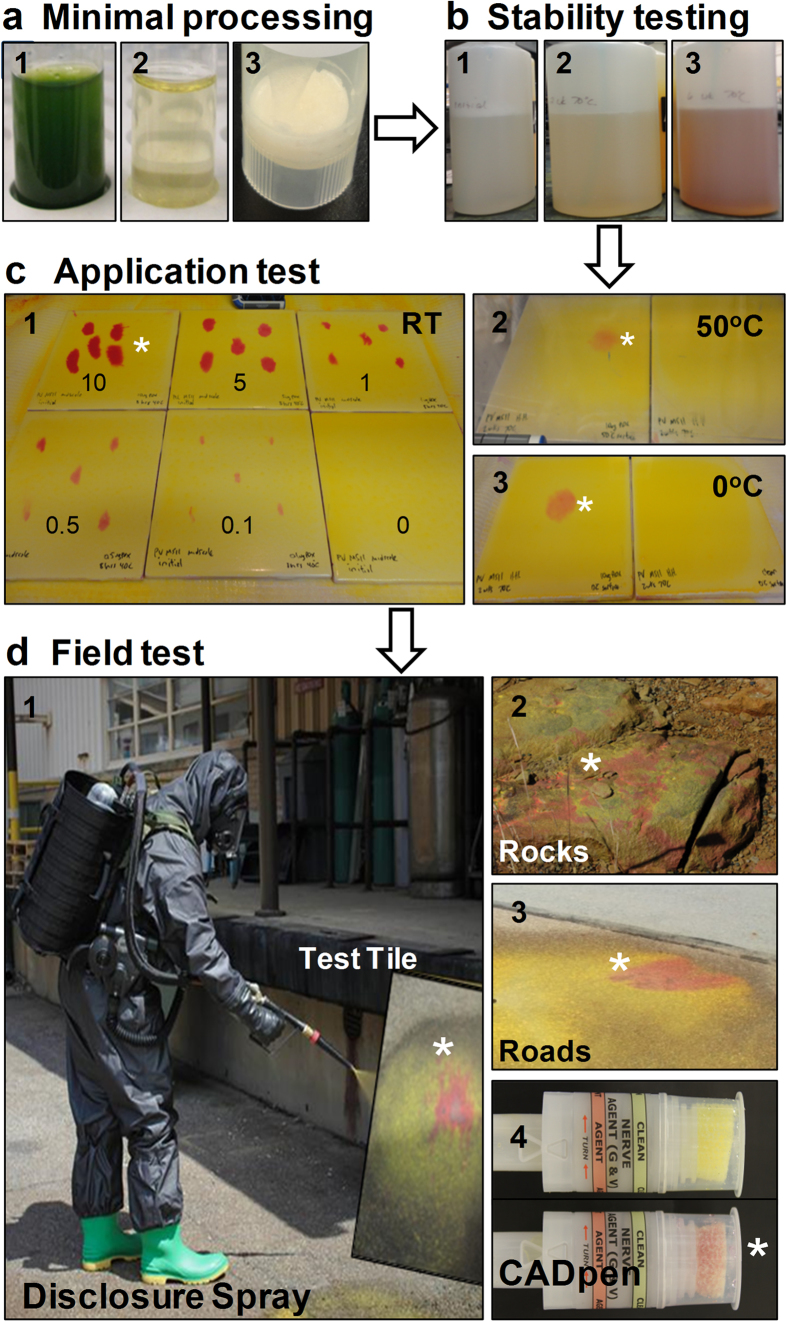
Key steps in the overall process in product development and modes of application of the final extract-based product. (**a**) Crude ground *N.benthamiana* leaf extract prior to centrifugation/filtration (a-1). Processed (clarified) leaf extract following removal of oils, fats and polyphenols using chitosan and PVP (a-2) Lyophilized leaf extract (a-3). (**b**) rHuAChE powder reconstituted either immediately (b-1) or following storage at 70 °C for 6 weeks with PVP (b-2) or without PVP (b-3). (**c**) Testing the enzymatic activity of a formulated spray containing the active reconstituted rHuAChE powder shown in 2b-2 against PX (0–10 ug) spotted on tiles previously stored at 23 °C (c-1), at 50 °C (c-2) and at 0 °C (c-3). The spots in panels C2 and C3 contain 10 ug of Px. Color changes from yellow to red, some examples of which are marked by white asterisks, indicate the presence of Px (see text). The number on each tile in c1 indicates the dose of Px (ug) used. (**d**) Field testing of the Disclosure spray on test tiles (d-1) on rocks (d-2) and roads (d-3). D4 shows an unused CAD sensor with clean sponge (top) and one that has come into contact with Px and changed to a red color (bottom).
